# Myelomeningocele: congenital neural tube defect anomaly (a rare clinical image)

**DOI:** 10.11604/pamj.2024.47.61.42654

**Published:** 2024-02-12

**Authors:** Shraddha Patil, Archana Mourya

**Affiliations:** 1 Department of Child Health Nursing, Smt. Radhikabai Meghe Memorial College of Nursing, Datta Meghe Institute of Higher Education and Research (Deemed University) Sawangi Wardha, Maharashtra, India

**Keywords:** Neural tube defect, neurosurgery, cyst, congenital defect, diagnosis

## Image in medicine

Myelomeningocele is a type of congenital birth defect that affects the spinal cord. It is the most severe form of spina bifida, it is a condition where the neural tube, which normally closes during fetal development to form the spine, fails to close properly. In myelomeningocele, the spinal cord and the nerves that control muscle function in the lower part of the body protrude through an opening in the baby's back. The spinal cord and the protective membranes around it (meninges) protrude through a hole in the vertebrae. That frequently results in a visible sac or bulge on the back, which is often covered by a thin layer of skin. Meningomyelocele, while considered a serious and potentially debilitating condition, is not extremely rare. It is a type of neural tube defect, and the frequency can vary among different populations and regions. The rare case seen in a 16-day-old newborn female was admitted to the outpatient department with a complaint of swelling in her lower back which gradually increased in size. The child was born with normal vaginal delivery with an average birth weight of 3kg. After a detailed examination and investigation, a myelomeningocele clinical diagnosis was made. The physician referred the child to a neonatal intensive care unit for further management and surgical intervention. The specific cause of meningomyelocele is not always known, but both genetic and environmental factors are believed to have a main role. Folic acid supplementation before and during early pregnancy has been instructed mother to minimize the incidence of neural tube abnormalities, therefore good diet and supplementation are essential components of prenatal care.

**Figure 1:**
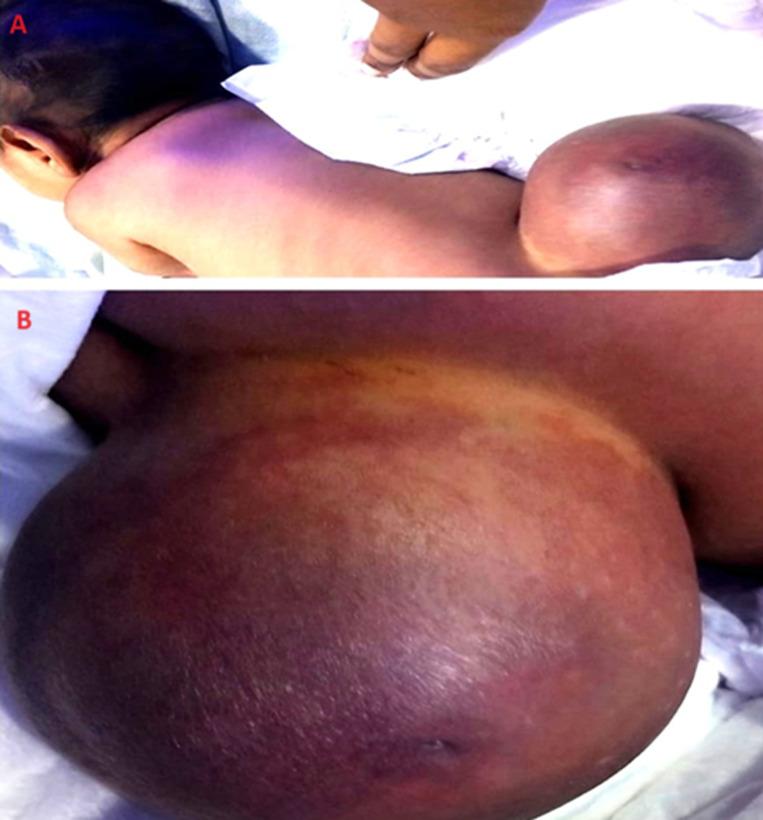
A, B) brownish colour abnormal sac present at the lower back region

